# Saunders’ Question Compends

**Published:** 1903-01

**Authors:** 


Saunders' Question Compends.—Essentials of Histology.—Second edition, revised and enlarged. By Louis Leroy, B. S., M. D., Professor of Histology and Pathology, Vanderbilt University, Medical and Dental Departments; Pathologist to the Nashville City Hospital, etc. Thoroughly revised and greatly enlarged. 16 mo. volume of 263 pages, with 92 beautiful illustrations. Philadelphia and London: W. B. Saunders & Co., 1902. Cloth, $1.00, net.

This little book contains the essentials of histology condensed into a very few pages and yet it is well adapted to serve as the means of refreshing one's memory in the details of the subject.

The illustrations are surprisingly good for so unpretentious a work. The chapter of technic is a valuable addition. It alone is worth more than the price charged for the entire book. R.

